# Mechanotransduction in Musculoskeletal Tissue Regeneration: Effects of Fluid Flow, Loading, and Cellular-Molecular Pathways

**DOI:** 10.1155/2014/863421

**Published:** 2014-08-18

**Authors:** Yi-Xian Qin, Minyi Hu

**Affiliations:** Department of Biomedical Engineering, Stony Brook University, Stony Brook, NY 11794-5281, USA

## Abstract

While mechanotransductive signal is proven essential for tissue regeneration, it is critical to determine specific cellular responses to such mechanical signals and the underlying mechanism. Dynamic fluid flow induced by mechanical loading has been shown to have the potential to regulate bone adaptation and mitigate bone loss. Mechanotransduction pathways are of great interests in elucidating how mechanical signals produce such observed effects, including reduced bone loss, increased bone formation, and osteogenic cell differentiation. The objective of this review is to develop a molecular understanding of the mechanotransduction processes in tissue regeneration, which may provide new insights into bone physiology. We discussed the potential for mechanical loading to induce dynamic bone fluid flow, regulation of bone adaptation, and optimization of stimulation parameters in various loading regimens. The potential for mechanical loading to regulate microcirculation is also discussed. Particularly, attention is allotted to the potential cellular and molecular pathways in response to loading, including osteocytes associated with Wnt signaling, elevation of marrow stem cells, and suppression of adipotic cells, as well as the roles of LRP5 and microRNA. These data and discussions highlight the complex yet highly coordinated process of mechanotransduction in bone tissue regeneration.

## 1. Introduction

High physical activity level has been associated with high bone mass and low fracture risk and is therefore recommended to reduce fractures [1–3]. The ability of musculoskeletal tissue to respond to changes in its functional milieu is one of the most intriguing aspects of such living tissue and certainly contributes to its success as a structure. Bone and muscle rapidly accommodate changes in their functional environment to ensure that sufficient skeletal mass is appropriately placed to withstand the regions of functional activity, an attribute described as Wolff's Law [4, 5]. This adaptive capability of musculoskeletal tissues suggests that biophysical stimuli may be able to provide a site-specific, exogenous treatment to control both bone mass and morphology. The premise of mechanical influence on bone morphology has become a basic tenet of bone physiology [6–8]. Absence of functional loading results in loss of bone mass [9–12], while exercise or increased activity results in increased bone mass [13–15]. Similarly, increasing exercise of musculoskeletal tissue can significantly increase blood flow, oxygen, and the exchange of fluid in muscle. During muscle contraction, several mechanisms regulate blood flow to ensure a close coupling between muscle oxygen delivery and metabolic demand [16–21]. Based on the muscle pump theory, vascular arteries and veins within skeletal muscles are compressed upon muscle contraction, therefore increasing the arteriovenous pressure gradient and promoting capillary filtration [22–24]. To define the formal relationship between mechanical milieu and the adaptive response, the relationship between muscle pump and interstitial fluid flow will prove instrumental in devising a mechanical intervention for musculoskeletal disorders such as osteoporosis, muscle fatigue, and atrophy, designing biomechanical means to accelerate fracture healing, and promoting bony ingrowth.

To adapt to the changing demands of mechanics, bone mass and bone morphology can be regulated via bone remodeling at specific sites. This crucial process of structural remodeling of the bone involves bone resorption and the subsequent bone formation. However, difficulties to determine specific mechanical components will hamper our understanding of bone remodeling related diseases, as well as limiting our judgments on bone fractures and healing capacity. Therefore, continuous studies of the bone remodeling process, for example, to determine the mechanical model of this remodeling process, can ultimately benefit the intervention on prevention and treatment of musculoskeletal disorders.

### 1.1. Bone Adaptation to Mechanical Loading

In the past few decades, researchers have suggested that the strain and stress are the main regulation parameters of bone cell response to mechanical signals. For instance, some researchers have proposed “invariant” parameter whose strength does not depend on a reference system, which is similar to “strain energy density” [25, 26] that is capable of modulating bone cell response to mechanical signals. This theory is consistent with the idea of bone self-regulation [27, 28]. There are many theories regarding bone self-regulation, including the degree of strain regulation on bone modeling process, time-dependent bone modeling, and remodeling processes [27]. A regulatory model with a variety of influential factors, including the magnitude of strain/stress, number of loading cycles, number of loading occurrences, tensor of strain, and the strain energy density, can result in bone self-regulation. It is still difficult to distinguish the independent effects of these factors and to determine the specific factors that regulate bone remodeling. To explore these mechanical hypotheses, it must be determined whether bone cells are directly or indirectly regulated by these mechanical parameters. By far, there is little evidence in the relationship between the maximal strain, stress, and bone morphology [9]. Specific mechanical parameters to initiate or discontinue mechanical response of bone cells remain to be further determined.

### 1.2. The Role of Dynamic and Temporal Mechanical Signals

**Figure 1:**
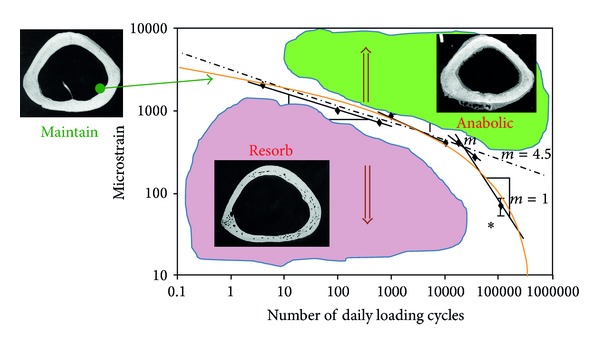
Maintaining bone mass as a function of daily loading cycle number requires a certain strain threshold (microstrain). A curve fitting to the data shows daily loading cycle numbers from less than one cycle to greater than 100,000 cycles. The necessary strain to maintain bone mass is reduced as the daily loading cycle number increases.

A recent discovery mainly uses the temporal portion of the stimulus signal, such as the number of strain cycles, loading frequency, and strain gradient, to explain the mechanism of bone response to mechanical stimuli at the cellular level. Under stimuli with the same strain magnitude, higher strain cycle will cause a more significant adaptive response [29, 30]. Similarly, signals at 15 to 60 Hz, in comparison to signals at about 1 Hz, can stimulate more bone growth [31, 32] (Figure 1). Maintenance of the existing bone mass requires different frequencies (1 to 60 Hz) of stimuli with continuous sinusoidal signal (10 minutes/day) to achieve different loading magnitude “threshold value.” Experiments have shown that stimulation at 1 Hz requires 700 *μ* *ε* of longitudinal strain to maintain the existing cortical bone mass, while stimulation at 30 Hz only requires 400 *μ* *ε*. If 60 Hz of the stimulation signal is used, only 270 *μ* *ε* of strain signal is sufficient to maintain the amount of cortical bone. A strong link has been found between this frequency-sensitive cortical bone remodeling process and the magnitude of bone fluid flow, in which the flow is directly regulated by the frequency (*R* = 0.8) [33]. Turner et al. have found that increased loading rate with a constant loading strain on the adult rat tibia can significantly improve bone formation [34]. Meanwhile, the amount of new bone formation is directly proportional to the strain loading rate. If we associate the external mechanical loading parameters with bone remodeling, then we will most likely be able to predict the periosteal new bone formation based on the strain gradient [9, 35, 36]. Many* in vivo* and* in vitro* experimental evidences have pointed out that bone adapts to dynamic mechanical loads rather than a static load [34, 37, 38]. All these show that dynamic and temporal mechanical signals, along with the potential load-induced fluid dynamics, are necessary for promoting the bone adaptation.

### 1.3. Mechanotransduction and Interstitial Bone Fluid Flow

Tensile strain is closely related to interstitial bone fluid flow caused by bone matrix deformation. Mechanical loading can cause variations of bone matrix deformation and interstitial fluid pathways within bone, thereby generating hydraulic pressure gradient within the capillary bed, leading to interstitial bone fluid flow [39]. Fluid flow-induced shear stress within bone has been considered as the source of how bone cells sense mechanical stimulation [40–44]. Bone interstitial fluid is filling a variety of voids and channels within the bone matrix, including lacunae-canaliculi, bone tubules, Haversian canal and Volkmann canal, and osteon [45]. Mechanical loading-induced interstitial bone fluid flow may play a role in mechanical sensing, bone cells response, signal transmission, transfer of nutrients, and so forth. This interstitial fluid flow within cortical bone is thought to be a critical regulator for bone mass and morphology [46–48]. We believe that this is a key mechanism of how bone, at certain loading frequency, strain, strain cycle, and strain gradient, leads to load-induced bone modeling, remodeling, and maintenance.

### 1.4. The Use of Noninvasive Dynamic Flow Stimulation to Prevent Bone Loss

Experiments on dogs during development have shown that increased venous pressure can promote new bone formation in the periosteum [49]. The data indicated that increased venous pressure will increase blood supply from the capillaries to the bone tissue, which may lead to new bone formation in the periosteum. In a rat tail suspension experiment, suturing tibial vein increased tibial marrow cavity pressure (ImP) (27.8 mmHg versus 16.4 mmHg, *P* < 0.05) in the experimental group compared to the control, which suggested that the pore fluid flow pressure reinforced by the suture is inversely proportional to the bone cross section. In the experimental group of vein suture, bone mineral content and trabecular bone density were significantly higher within 19 days [50]. These results indicated that bone fluid flow may not solely rely on the mechanical loads to cause bone adaptive response. Moreover, intravenous fluid pressure is directly related to hydraulic ImP, which implies that the adaptive response can be altered by intravenous bone muscle pump hydraulic effect that prevents bone loss noninvasively.

### 1.5. Marrow Pressure and Bone Strain Generated by Mechanical Loading

Recent studies have revealed that induced marrow fluid pressure and bone strain by mechanical stimulation were dependent on dynamic loading parameters and optimized at certain loading frequencies [33]. A previous study has evaluated oscillatory electrical muscle stimulation- (MS-) induced ImP and bone strain as function of stimulation frequency. MS generated femoral ImP and bone strain were measured with frequencies of 1–100 Hz in rats. A maximum ImP of 45 ± 9 mmHg at 20 Hz and a maximum matrix strain of 128 ± 19 *μ* *ε* at 10 Hz were generated by oscillatory MS. These results suggest that muscle force alone, if applied at a low rate, such as resistant weight lifting with high intensity, would not be able to generate sufficient strain and fluid pressure in bone. MS with a relatively high rate and a small magnitude, however, can trigger significant fluid pressure in the skeleton. To identify induced ImP dynamics and bone strain factors* in vivo* using a noninvasive method, a more recent study used dynamic hydraulic stimulation (DHS) and evaluated its immediate effects on local and distant ImP and bone strain in response to a range of loading frequencies of 1 Hz to 10 Hz [51–54]. DHS-induced ImP in the stimulated tibia was in a nonlinear fashion over the range of loading frequencies, where they peaked at 2 Hz with a maximum ImP of 14.48 ± 3.10 mmHg. Maximal bone strain was less than 8 *μ* *ε*, measured at all loading frequencies. No detectable induction of ImP or bone strain was observed in the distant site away from the stimulation. Oscillatory DHS may regulate local fluid dynamics with minimal mechanical matrix strain, which is highly frequency dependent.

### 1.6. Mechanical Loading-Induced Bone Loss Attenuation and Fracture Healing

**Figure 2:**
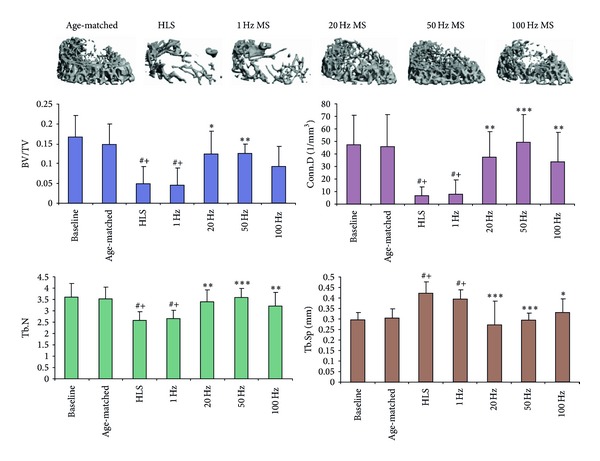
Representative 3D *μ*CT images of trabecular bone in distal femur. Graphs show mean ± SD values for bone volume fraction (BV/TV, %), connectivity density (Conn.D, 1/mm^3^), trabecular number (Tb.N, 1/mm), and separation (Tb.Sp, mm). MS at 50 Hz produced a significant change in all indices, compared to HLS. ^#^ *P* < 0.001 versus baseline; ^+^ *P* < 0.001 versus age-matched; *P < 0.05 versus HLS and 1 Hz MS; **P < 0.01 versus HLS and 1 Hz MS; ***P < 0.001 versus HLS and 1 Hz MS.

**Figure 3:**
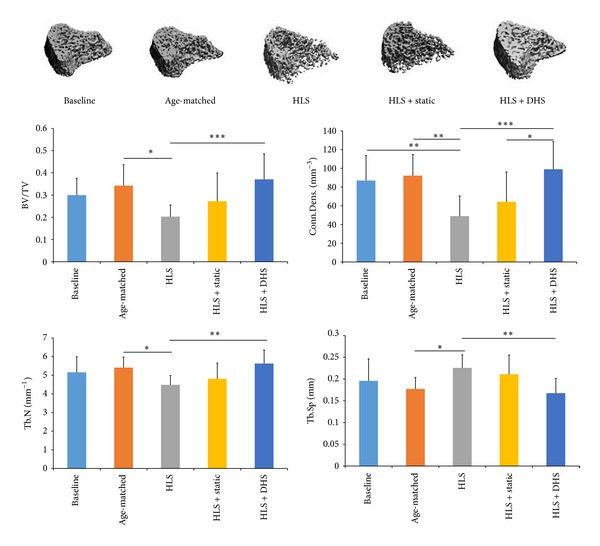
Representative 3D *μ*CT images of trabecular bone in distal femur. Graphs show mean ± SD values for bone volume fraction (BV/TV, %), connectivity density (Conn.D, 1/mm^3^), trabecular number (Tb.N, 1/mm), and separation (Tb.Sp, mm). DHS at 2 Hz produced a significant change in all indices, compared to HLS. *P < 0.05; **P < 0.01; ***P < 0.001.

An* in vivo* study used a rat functional disuse model to evaluate the mitigation potential of MS in disused trabecular bone and investigated the importance of the optimized stimulation frequency (1, 20, 50, and 100 Hz) in the loading regimen [52]. Analyzed by microCT and histomorphometry, MS for 10 min/day with a total of 4 weeks showed improvements in metaphyseal trabecular bone quantity and structure at midfrequency (20 Hz and 50 Hz), in which 50 Hz of stimulation demonstrated the greatest preventive effect on the skeleton against functional disuse (up to +147% in bone volume fraction, +38% in trabecular number, and −36% in trabecular separation compared to HLS control). These data imply that MS, applied at a high frequency with a low magnitude for a short duration, is able to mitigate bone loss induced by the functional disuse (Figure 2). In addition, another study used DHS that elevates* in vivo* oscillatory BFF via ImP, to evaluate the effects of DHS on mitigation of trabecular bone loss and structural alteration in a rat disuse model [53–56]. DHS of 2 Hz for 20 min/day, 5 days/week, and a total of 4-week experiment improved the bone quantity and microarchitecture (+83% in bone volume fraction, +25% in trabecular number, and −26% in trabecular separation compared to HLS control). The data demonstrate DHS's potentials to mitigate bone loss induced by functional disuse (Figure 3).

Taking into account that MS can increase blood flow and ImP in the muscle and marrow cavity [33, 57] and that blood flow has a close relationship with fracture healing, it is likely that applying MS may result in an enhancement of fracture healing. Using a rabbit model with a 3 mm tibial transverse osteotomy, Park and Silva have shown that fracture treated with MS showed 31% higher mineral content and 27% larger callus area than control osteotomies at eight weeks. In addition, the maximum torque, torsional stiffness, angular displacement at maximum torque, and energy required for failure of specimens in the study group were 62%, 29%, 34.6%, and 124% higher, respectively, compared to the control at eight weeks [58]. The results suggested that the use of MS can enhance callus mineralization and biomechanical strength in the callus region. This may, at least partially, be the result of MS enhanced blood circulation. Using a bone chamber, Winet and his group observed that muscle contractions directly increased bone blood flow rates by 130% but uncoupled from mechanical loading, while heart rates and blood pressure did not significantly increase due to the MS treatment [59]. Thus, enhanced fluid flow by MS may directly involve increasing fluid flow in callus and trigger anabolic response under such acute conditions, for example, fracture healing.

## 2. Potential Cellular and Molecular Pathways of Mechanotransduction

Bone remodeling involves all related cell types, that is, osteoblast, osteoclast, osteocyte, T-cells, B-cells, megakaryocyte, and lining cells. Thus, all these cells are potentially mechanosensitive and even interrelated. These cells respond to mechanical loading with expression of specific molecular pathways. This section will discuss several potential pathways involved in mechanical stimulation induced adaptation.

### 2.1. Basic Multicellular Units (BMU)

**Figure 4:**
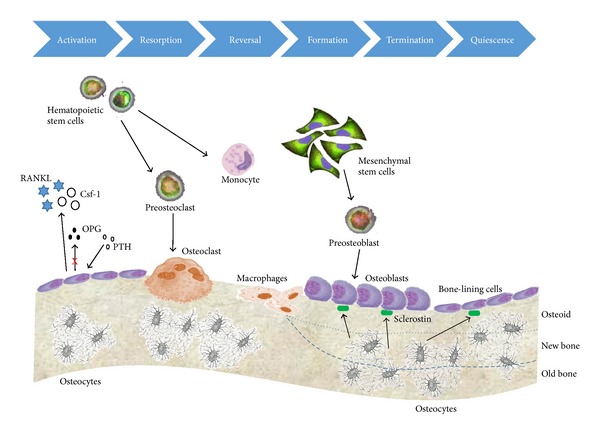
Bone remodeling and its associated molecular pathways. The remodeling cycle of bone is composed of sequential phases including the activation of precursor cells, bone resorption by osteoclasts, bone formation by osteoblasts after reversal, and mineralization. The osteoblasts that are buried within the newly formed matrix become osteocytes. Other osteoblasts that rest on the bone surface become bone-lining cells.

To explore the interrelation among overall bone cells, a cluster of bone forming and bone resorption cells among dynamic and temporal adaptation structures are known as “basic multicellular units” (BMUs) [60, 61]. Bone adaptation occurs constantly and each cycle may take over several weeks. Such processes are performed with combination of resorption and formation. Each phase can involve targeted molecular and gene activations. An active BMU consists of a leading front of bone-resorbing osteoclasts. Reversal cells, of unclear phenotype, follow the osteoclasts, covering the newly exposed bone surface, and prepare them for deposition of replacement bone, following a deposition of an unmineralized bone matrix known as osteoid. Related molecular and genetic factors are represented in this temporal sequence (Figure 4).

In response to mechanical loading, the first stage of remodeling reflects the detection of initiating triggering signals such as fluid flow and/or any other physical stimulation, for example, pressure, electrical, and acoustic waves. Prior to activation, the resting bone surface is covered with bone-lining cells, including preosteoblasts intercalated with osteomacs. B-cells are present in the bone marrow and secrete osteoprotegerin (OPG) that suppresses osteoclastogenesis. During the activation phase, the endocrine bone remodeling signal parathyroid hormone (PTH) binds to the PTH receptor on preosteoblasts. Damage to the mineralized bone matrix results in localized osteocyte apoptosis, reducing the local transforming growth factor *β* (TGF-*β*) concentration and its inhibition of osteoclastogenesis. In the resorption phase, in response to PTH signaling, MCP-1 is released from osteoblasts and recruits preosteoclasts to the bone surface. Additionally, osteoblastic expression of OPG is decreased, and production of CSF-1 and RANKL is increased to promote proliferation of osteoclast precursors and differentiation of mature osteoclasts. Mature osteoclasts anchor to RGD-binding sites, creating a localized microenvironment (sealed zone) that facilitates degradation of the mineralized bone matrix. In the reversal phase, reversal cells engulf and remove demineralized undigested collagen from the bone surface. Transition signals are generated that halt bone resorption and stimulate the bone formation process. During the formation phase, formation signals and molecules arise from the degraded bone matrix, mature osteoclasts, and potentially reversal cells. PTH and mechanical activation of osteocytes reduce sclerostin expression, allowing for Wnt-directed bone formation to occur. Finally, in the termination phase, sclerostin expression likely returns, and bone formation ceases. The newly deposited osteoid is mineralized; the bone surfaces return to a resting state with bone-lining cells intercalated with osteomacs, and the remodeling cycle ends. Mechanical stimulation is likely involved in each of these phases and eventually regulates related molecular and genetic factors. This unique spatial and temporal arrangement of cells within the BMU is critical to bone remodeling, ensuring coordination of the distinct and sequential phases of this process: activation, resorption, reversal, formation, and termination.

### 2.2. Osteocyte and Its Response to Mechanical Signals Coupled with Wnt Signaling

**Figure 5:**
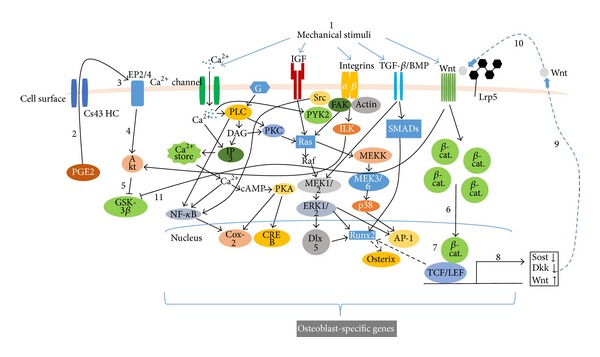
Mechanical stimulation activates intracellular signaling pathways that converge with growth factors to activate transcription factors, which promotes bone formation. Perception of load (strain, “1”) triggers a number of intracellular responses including the release of PGE2, “2,” through a poorly understood mechanism into the lacunar-canalicular fluid where it can act in an autocrine and/or paracrine fashion. Connexin-43 hemichannels (CX43 HC) in this PGE2 and integrin proteins appear to be involved. Binding of PGE2 to its EP2 and/or EP4 receptor, “3,” leads to a downstream inhibition of GSK-3*β*, “5” (likely mediated by Akt, “4”) and the intracellular accumulation of free *β*-catenin, “6.” (Integrin activation can also lead to Akt activation and GSK-3*β* inhibition.) New evidence suggests that ER may participate in the nuclear translocation of *β*-catenin, “7,” which leads to changes in the expression of a number of key target genes “8.” One of the apparent consequences is the reduction in sclerostin and Dkk1, “9,” with increased expression of Wnt, “10”. The net result of these changes is to create a permissive environment for the binding of Wnt to LRP5-Fz and an amplification of the load signal, “11.”

Osteocytes, cells embedded within the mineralized matrix of bone, are becoming the target of intensive investigation [61–64]. Osteoblasts are defined as cells that make bone matrix and are thought to translate mechanical loading into biochemical signals that affect bone modeling and remodeling. The interrelationship between osteoblasts and osteocytes would be expected to have the same lineage, yet these cells also have distinct differences, particularly in their responses to mechanical loading and utilization of the various biochemical pathways to accomplish their respective functions. Among many factors, Wnt/*β*-catenin signaling pathway may be recognized as an important regulator of bone mass and bone cell functions [61, 64]. While osteocytes are embedded within the mineral matrix, Wnt/*β*-catenin signaling pathway may serve as a transmitter to transfer mechanical signals sensed by osteocytes to the surface of bone. Further, new data suggest that the Wnt/*β*-catenin pathway in osteocytes may be triggered by crosstalk with the prostaglandin pathway in response to loading which then leads to a decrease in expression of negative regulators of the pathway such as sclerostin (Sost) and Dickkopf-related protein 1 (Dkk-1) [64, 65].  Figure 5 indicates potential pathway in response to mechanical loading.

It has been shown that the Wnt pathway is closely involved in bone cell differentiation, proliferation, and apoptosis [64, 66]. Regulation of the Wnt/*β*-catenin signaling pathway is vested largely in proteins that either act as competitive binders of Wnts, notably the secreted frizzled-related proteins (sFRP) family, or act at the level of low-density lipoprotein receptor-related protein 5 (LRP5), including the osteocyte specific protein, sclerostin (the Sost gene product), and the Dkk proteins, particularly Dkk-1 and Dkk-2 [64, 66–69]. Sclerostin has been shown to be made by mature osteocytes and inhibits Wnt/*β*-catenin signaling by binding to LRP5 and preventing the binding of Wnt. Dkk-1 is highly expressed in osteocytes [67–69]. Clinical trial studies using antibodies to sclerostin have also been shown to result in increased bone mass, suggesting that targeting of these negative regulators of Wnt/*β*-catenin signaling pathway might be anabolic treatments for diseases such as osteoporosis [67]. Finally, mechanical loading has been shown to reduce sclerostin levels in bone [67], suggesting that one of the targets of the pathways, activated by the early events after mechanical loading, is the genes encoding these negative modulators of the Wnt/*β*-catenin signaling pathway.

### 2.3. Mechanical Signal Triggered Bone Marrow Cells Alteration

The data from disuse osteopenia and clinical osteoporosis have shown significant reduction of bone density and structural integrity, culminating in an elevated risk of skeletal fracture. Concurrently, a marked reduction in the available bone-marrow-derived population of mesenchymal stem cells (MSCs) [70] jeopardizes the regenerative potential that is critical to the recovery from bone loss, musculoskeletal injury, and diseases. A potential way to combat the deterioration involves harnessing the sensitivity of bone to mechanical signals, which is crucial in defining, maintaining, and recovering bone mass. As discussed above, bone cells, that is, osteoblast, osteoclast, and osteocyte, may sense external mechanical loading directly and perform balance of formation and resorption in the remodeling process; specific mechanotransductive signals may also bias MSC differentiation towards osteoblastogenesis and away from adipogenesis. Mechanical targeting of the bone marrow stem-cell pool might, therefore, represent a novel, drug-free means of slowing the age-related decline of the musculoskeletal system.

Exercise is important in stemming both osteoporosis and obesity, with the fact that MSCs are progenitors of both osteoblasts and adipocytes (fat cells), as well as the anabolic response of the skeletal system to mechanical loadings. It was then hypothesized that mechanical signals anabolic to bone would invariably cause a parallel decrease in fat production. In an* in vivo* setting, seven-week-old C57BL/6J mice on a normal chow diet were randomized to undergo low magnitude high frequency loading (90 Hz at 0.2 g for 15 minutes per day) or placebo treatment [71]. At 15 weeks, with no differences in food consumption between groups,* in vivo* CT scans showed that the abdominal fat volume of mice subjected to loading was 27% lower than that of the controls (*P* < 0.01) [72, 73]. Wet weights of visceral and subcutaneous fat deposits in loading mice were correspondingly lower. Confirmed by fluorescent labeling and flow cytometry studies [72, 73], these data indicated that the influence of mechanical signals is not only on the resident bone cell (osteoblast/osteocyte) population but also on their progenitors, biasing MSC differentiation towards bone (osteoblastogenesis) and away from fat (adipogenesis). In a follow-up test of this hypothesis, mice fed with a high fat diet were subjected to low magnitude loading or placebo treatment [72, 73]. Suppression of adiposity by the mechanical signals was accompanied by a “mechanistic response” at the molecular level, which shows that loading significantly influenced MSC commitment to either osteogenic runt-related transcription factor 2 (Runx2), a transcription factor central to osteoblastogenesis, or adipogenic (peroxisome proliferator-activated receptor [PPAR]*γ*, a transcription factor central to adipogenesis). Runx2 expression was greater and PPAR*γ* expression was decreased in the mice that underwent LMMs compared with controls. The PPAR*γ* transcription factor, when absent or present as a single copy, facilitates osteogenesis at least partly through enhanced canonical Wnt signaling [74, 75], a pathway critically important to MSC entry into the osteogenic lineage and expansion of the osteoprogenitor pool. Notably, low magnitude mechanical loading treatment also resulted in a 46% increase in the size of the MSC pool (*P* < 0.05) [72, 73]. These experiments, although not obviating a role for the osteoblast/osteocyte syncytium, provide evidence that bone marrow stem cells are capable of sensing exogenous mechanical signals and responding with an alteration in the cell fate that ultimately influences both the bone and fat phenotypes. Importantly, the inverse correlation of bone and fat phenotypes has increasing support in the clinical literature. Although controversial, and despite the presumption that conditions such as obesity will inherently protect the skeleton owing to increased loading events, data in humans evaluating bone-fat interactions indicate that an ever-increasing adipose burden comes at the cost of bone structure and increased risk of fracture [76].

### 2.4. The Role of LRP5 in Bone Responding to Mechanical Loading

LRP5 has been shown to have important functions in the mammalian skeleton. Experimental evidences have pointed LRP5 as a critical factor in translating mechanical signals into the proper skeletal response. For example, loss-of-function mutations in LRP5 have been reported to cause the autosomal recessive human disease osteoporosis-pseudoglioma syndrome (OPPG), which leads to significant reduction of BMDs, and are more susceptible to skeletal fracture and deformity [77–79]. Moreover, the mechanical importance of LRP5 has been demonstrated in LRP5−/− mice, which were found with an almost complete ablation in ulnar loading-induced bone formation compared to wild-type controls [79, 80]. Multiple single nucleotide polymorphisms (SNPs), located in exons 18 and 10, have been reported, which can significantly affect the interconnection between physical activity and bone mass [79, 81]. A high bone mass (HBM) phenotype in humans was reported to be caused by certain missense mutations near the N-terminus of LRP5 [82, 83]. An LRP5 overexpression mutation is, on the other hand, associated with high bone mass and induced osteoblast proliferation [82]. Increased sensitivity to load due to a lower threshold for initiating bone formation was also reported with this mouse [83]. A recent study done by Zhong et al. showed that* in vitro* tension on MC3T3-E1 cells increased LRP5 gene expression at 1, 3, and 5 hours of loading [84].

### 2.5. MicroRNA and Its Role in Mechanotransduction in Tissue

The newly discovered microRNAs (miRNAs) are short noncoding RNAs, which can be complementary to messenger RNA (mRNA) sequences to silent gene expression by either degradation or inhibitory translation of target transcripts [85, 86]. Regulation of Runx2, bone morphogenic protein (BMP), and Wnt signaling pathways is by far the most well-studied miRNA related osteoblast function. Positive and negative regulations of miRNAs on Runx2 expression have been shown to affect skeletal morphogenesis and osteoblastogenesis [87]. Inhibition of osteoblastogenesis can result from miRNA-135 and miRNA-26a regulated BMP-2/Smad signaling pathway [88]. Activation of Wnt signaling through miRNA-29a-targeted Wnt inhibitors is upregulated during osteoblast differentiation [89]. In addition, studies have been done to investigate the miRNA function on self-renewal and lineage determination for tissue regeneration via human stromal stem cells [90, 91]. Moreover, extensive studies have also been done to assess the effects of miRNAs on osteogenic functions in committed cell lines including osteoprogenitors, osteoblasts, and osteocytic cell lines. In general, actions of miRNA may affect bone cell differentiation in either positive or negative ways [85, 91].

Recent research has gained interests in studying the transcription and microRNA regulation to better understand gene expression regulation in a mechanical loading model. Transcription factors can bind to motifs in the promoter of genes and directly affect their expression; therefore, mechanotransduction in bone may result in transcription factors alteration for regulation. Using a predictive bioinformatics algorithm, a recent study investigated the time-dependent regulatory mechanisms that governed mechanical loading-induced gene expression in bone. Axial loading was performed on the right forelimb in rodents. A linear model of gene expression was created and 44 transcription factor binding motifs and 29 microRNA binding sites were identified to predict the regulated gene expression across the time course. It may be important in controlling the loading-induced bone formation process via the time-dependent regulatory mechanisms.

### 2.6. Mechanotransductive Implication in Bone Tissue Engineering

Development of artificial scaffold for musculoskeletal applications could take advantage of the mechanotransduction phenomena to achieve its integrity and function, which can lead to tissue healing. Mechanical signals delivered to bone cells may be interfered by the scaffold deformation and should be taken into account. Fortunately, mechanotransduction could be used to control the proliferation and differentiation of bone cells [55, 56, 92–94]. Fluid flow has been proposed as an important mechanical aspect to be considered when developing bone scaffolds [53–56, 94]. Studies using bioreactors have helped us understand the phenomena of mechanotransduction used in scaffold design [92]. For example, rotating bioreactors, flow perfusion bioreactors, and other mechanical stimuli such as strain have been designed to increase mass transfer by inducing dynamic flow conditions in culture, to create osteoinductive factors on mesenchymal stem cells by the generated fluid shear stress [95], and to induce the osteogenic differentiation of mesenchymal stem cells [96], respectively. Among all, mimicking the natural bone strain to favor osteogenesis is one of the most rational aims for scaffold development. Matching of the strain histograms of a scaffold and the actual bone can be performed using microCT measurements and finite element method [55, 97, 98].

## 3. Summary

Functional tissue regeneration has been shown to be significantly influenced by mechanical loading and mechanotransduction under both* in vitro* and* in vivo* conditions. There are close interrelationships among bone, muscle, cellular, molecular pathways, and biomaterial remodeling by such physiological stimulation. The effects of mechanobiology may be harnessed in such a way that dynamic fluid flow stimulation can act as a mechanobiological mediator in scaffold to regulate cellular and tissue regeneration and proliferation. Such signals must be performed and conducted in a dynamic manner and potentially served as a noninvasive approach. The increase of physiological stimulation may ultimately enhance interstitial fluid flow and mechanotransduction in tissue and engineered constructs. Furthermore, dynamic stimulation, if applied at an optimal frequency, has shown the potential to attenuate osteopenia in disuse while promoting formation in osteogenesis, which may potentially serve as a biomechanical intervention for treating osteoporosis and muscle atrophy.

## References

[1] Gerdhem P, Ringsberg KAM, Åkesson K, Obrant KJ (2003). Influence of muscle strength, physical activity and weight on bone mass in a population-based sample of 1004 elderly women. *Osteoporosis International*.

[2] Gerdhem P, Ringsberg KAM, Magnusson H, Obrant KJ, Åkesson K (2003). Bone mass cannot be predicted by estimations of frailty in elderly ambulatory women. *Gerontology*.

[3] LeBlanc A, Lin C, Shackelford L (2000). Muscle volume, MRI relaxation times (T2), and body composition after spaceflight. *Journal of Applied Physiology*.

[4] Wolff J (1892). *Das Gesetz der Transformation der Knochen*.

[5] Wolff J (1986). *The Law of Bone Remodeling*.

[6] Evans FG, Vincentelli R (1974). Relations of the compressive properties of human cortical bone to histological structure and calcification. *Journal of Biomechanics*.

[7] Martin RB, Burr DB (1989). *Structure, Function and Adaptation of Compact Bone*.

[8] Stokes IA (1997). Analysis of symmetry of vertebral body loading consequent to lateral spinal curvature. *Spine*.

[9] Gross TS, Edwards JL, Mcleod KJ, Rubin CT (1997). Strain gradients correlate with sites of periosteal bone formation. *Journal of Bone and Mineral Research*.

[10] Qin YX, Otter MW, Rubin CT, McLeod KJ (1997). The influence of intramedullary hydrostatic pressure on transcortical fluid flow patterns in bone. *Transactions of the Annual Meeting of the Orthopaedic Research Society*.

[11] Rubin CT, Lanyon LE (1984). Regulation of bone formation by applied dynamic loads. *Journal of Bone and Joint Surgery A*.

[12] Turner CH (1999). Site-specific skeletal effects of exercise: importance of interstitial fluid pressure. *Bone*.

[13] Jones HH, Priest JD, Hayes WC, Tichenor CC, Nagel DA (1977). Humeral hypertrophy in response to exercise. *Journal of Bone and Joint Surgery A*.

[14] Krolner B, Toft B, Nielsen SP, Tondevold E (1983). Physical exercise as prophylaxis against involutional vertebral bone loss: a controlled trial. *Clinical Science*.

[15] Nilsson BE, Westlin NE (1971). Bone density in athletes. *Clinical Orthopaedics and Related Research*.

[16] Joyner MJ, Proctor DN (1999). Muscle blood flow during exercise: the limits of reductionism. *Medicine and Science in Sports and Exercise*.

[17] Joyner MJ (2001). Blood pressure and exercise: Failing the acid test. *Journal of Physiology*.

[18] Hicks A, McGill S, Hughson RL (1999). Tissue oxygenation by near-infrared spectroscopy and muscle blood flow during isometric contractions of the forearm. *Canadian Journal of Applied Physiology*.

[19] Mayet-Sornay MH, Hoppeler H, Shenkman BS, Desplanches D (2000). Structural changes in arm muscles after microgravity. *Journal of Gravitational Physiology*.

[20] Serova LV (2001). Microgravity and aging of animals. *Journal of Gravitational Physiology*.

[21] Convertino VA (2002). Mechanisms of microgravity induced orthostatic intolerance: implications for effective countermeasures. *Journal of Gravitational Physiology*.

[22] Laughlin MH (2005). The muscle pump: what question do we want to answer?. *Journal of Applied Physiology*.

[23] Otter MW, Qin YX, Rubin CT, McLeod KJ (1999). Does bone perfusion/reperfusion initiate bone remodeling and the stress fracture syndrome?. *Medical Hypotheses*.

[24] Winet H, Noble B, Jones D (2003). A bone fluid flow hypothesis for muscle pump-driven capillary filtration: II Proposed role for exercise in erodible scaffold implant incorporation. *European Cells and Materials*.

[25] Fyhrie DP, Carter DR (1986). A unifying principle relating stress to trabecular bone morphology. *Journal of Orthopaedic Research*.

[26] Huiskes R, Weinans H, Grootenboer HJ, Dalstra M, Fudala B, Slooff TJ (1987). Adaptive bone-remodeling theory applied to prosthetic-design analysis. *Journal of Biomechanics*.

[27] Frost HM (1986). *Intermediary Organization of the Skeleton*.

[28] Rubin CT, Gross TS, Mcleod KJ, Bain SD (1995). Morphologic stages in lamellar bone formation stimulated by a potent mechanical stimulus. *Journal of Bone and Mineral Research*.

[29] Goldstein SA, Mattews LS, Kuhn JL, Hollister SJ (1991). Trabecular bone remodeling: an experimental model. *Journal of Biomechanics*.

[30] Richards M, Kozloff KM, Goulet JA, Goldstein SA (2000). Increased distraction rates influence precursor tissue composition without affecting bone regeneration. *Journal of Bone and Mineral Research*.

[31] McLeod K, Rubin CT (1992). Sensitivity of the bone remodeling response to the frequency of applied strain. *Transactions of the Orthopaedic Research Society*.

[32] Qin Y-X, Rubin CT, McLeod KJ (1998). Nonlinear dependence of loading intensity and cycle number in the maintenance of bone mass and morphology. *Journal of Orthopaedic Research*.

[33] Qin Y, Lam H (2009). Intramedullary pressure and matrix strain induced by oscillatory skeletal muscle stimulation and its potential in adaptation. *Journal of Biomechanics*.

[34] Turner CH, Owan I, Takano Y (1995). Mechanotransduction in bone: role of strain rate. *The American Journal of Physiology—Endocrinology and Metabolism*.

[35] Judex S, Gross TS, Zernicke RF (1997). Strain gradients correlate with sites of exercise-induced bone-forming surfaces in the adult skeleton. *Journal of Bone and Mineral Research*.

[36] Judex S, Zernicke RF (2000). High-impact exercise and growing bone: relation between high strain rates and enhanced bone formation. *Journal of Applied Physiology*.

[37] Robling AG, Duijvelaar KM, Geevers JV, Ohashi N, Turner CH (2001). Modulation of appositional and longitudinal bone growth in the rat ulna by applied static and dynamic force. *Bone*.

[38] Rubin CT, McLeod KJ (1994). Promotion of bony ingrowth by frequency-specific, low-amplitude mechanical strain. *Clinical Orthopaedics and Related Research*.

[39] Piekarski K, Munro M (1977). Transport mechanism operating between blood supply and osteocytes in long bones. *Nature*.

[40] Reich KM, Gay CV, Frangos JA (1990). Fluid shear stress as a mediator of osteoblast cyclic adenosine monophosphate production. *Journal of Cellular Physiology*.

[41] Turner CH, Forwood MR, Otter MW (1994). Mechanotransduction in bone: do bone cells act as sensors of fluid flow?. *The FASEB Journal*.

[42] Weinbaum S, Cowin SC, Zeng Y (1994). A model for the excitation of osteocytes by mechanical loading-induced bone fluid shear stresses. *Journal of Biomechanics*.

[43] Weinbaum S, Guo P, You L (2001). A new view of mechanotransduction and strain amplification in cells with microvilli and cell processes. *Biorheology*.

[44] You J, Yellowley CE, Donahue HJ, Zhang Y, Chen Q, Jacobs CR (2000). Substrate deformation levels associated with routine physical activity are less stimulatory to bone cells relative to loading-induced oscillatory fluid flow. *Journal of Biomechanical Engineering*.

[45] Pollack SR, Salzstein R, Pienkowski D (1984). Streaming potential in fluid filled bone. *Ferroelectrics*.

[46] Montgomery RJ, Sutker BD, Bronk JT, Smith SR, Kelly PJ (1988). Interstitial fluid flow in cortical bone. *Microvascular Research*.

[47] Salzstein RA, Pollack SR (1987). Electromechanical potentials in cortical bone: II. Experimental analysis. *Journal of Biomechanics*.

[48] Skripitz R, Aspenberg P (2000). Pressure-induced periprosthetic osteolysis: a rat model. *Journal of Orthopaedic Research*.

[49] Kelly PJ, Bronk JT (1990). Venous pressure and bone formation. *Microvascular Research*.

[50] Bergula AP, Huang W, Frangos JA (1999). Femoral vein ligation increases bone mass in the hindlimb suspended rat. *Bone*.

[51] Hu M, Qin YX (2014). Dynamic fluid flow stimulation on cortical bone and alterations of the gene expressions of osteogenic growth factors and transcription factors in a rat functional disuse model. *Archives of Biochemistry and Biophysics*.

[52] Lam H, Qin Y (2008). The effects of frequency-dependent dynamic muscle stimulation on inhibition of trabecular bone loss in a disuse model. *Bone*.

[53] Qin YX, McLeod K, Rubin CT (1999). Intramedullary pressure indued fluid flow in bone. *Annals of Biomedical Engineering*.

[54] Qin Y, Lam H, Ferreri S, Rubin C (2010). Dynamic skeletal muscle stimulation and its potential in bone adaptation. *Journal of Musculoskeletal Neuronal Interactions*.

[55] Ferreri SL, Talish R, Trandafir T, Qin Y (2011). Mitigation of bone loss with ultrasound induced dynamic mechanical signals in an OVX induced rat model of osteopenia. *Bone*.

[56] Hu M, Cheng J, Qin YX (2012). Dynamic hydraulic flow stimulation on mitigation of trabecular bone loss in a rat functional disuse model. *Bone*.

[57] Sherry JE, Oehrlein KM, Hegge KS, Morgan BJ (2001). Effect of burst-mode transcutaneous electrical nerve stimulation on peripheral vascular resistance. *Physical Therapy*.

[58] Park S, Silva M (2004). Neuromuscular electrical stimulation enhances fracture healing: results of an animal model. *Journal of Orthopaedic Research*.

[59] Caulkins C, Ebramzadeh E, Winet H (2009). Skeletal muscle contractions uncoupled from gravitational loading directly increase cortical bone blood flow rates in vivo. *Journal of Orthopaedic Research*.

[60] Sims NA, Martin TJ (2014). Coupling the activities of bone formation and resorption: a multitude of signals within the basic multicellular unit. *BoneKEy Reports*.

[61] Raggatt LJ, Partridge NC (2010). Cellular and molecular mechanisms of bone remodeling. *Journal of Biological Chemistry*.

[62] Bonewald LF (2002). Osteocytes: a proposed multifunctional bone cell. *Journal of Musculoskeletal Neuronal Interactions*.

[63] Bonewald LF (2007). Osteocytes as dynamic multifunctional cells. *Annals of the New York Academy of Sciences*.

[64] Bonewald LF, Johnson ML (2008). Osteocytes, mechanosensing and Wnt signaling. *Bone*.

[65] Armstrong VJ, Muzylak M, Sunters A (2007). Wnt/beta-catenin signaling is a component of osteoblastic bone cell early responses to load-bearing and requires estrogen receptor α. *Journal of Biological Chemistry*.

[66] Robinson JA, Chatterjee-Kishore M, Yaworsky PJ (2006). Wnt/beta-catenin signaling is a normal physiological response to mechanical loading in bone. *Journal of Biological Chemistry*.

[67] Ke HZ, Richards WG, Li X, Ominsky MS (2012). Sclerostin and dickkopf-1 as therapeutic targets in bone diseases. *Endocrine Reviews*.

[68] Li X, Zhang Y, Kang H (2005). Sclerostin binds to LRP5/6 and antagonizes canonical Wnt signaling. *Journal of Biological Chemistry*.

[69] Li X, Liu P, Liu W (2005). Dkk2 has a role in terminal osteoblast differentiation and mineralized matrix formation. *Nature Genetics*.

[70] Ozcivici E, Luu YK, Adler B (2010). Mechanical signals as anabolic agents in bone. *Nature Reviews Rheumatology*.

[71] Rubin CT, Capilla E, Luu YK (2007). Adipogenesis is inhibited by brief, daily exposure to high-frequency, extremely low-magnitude mechanical signals. *Proceedings of the National Academy of Sciences of the United States of America*.

[72] Luu YK, Capilla E, Rosen CJ (2009). Mechanical stimulation of mesenchymal stem cell proliferation and differentiation promotes osteogenesis while preventing dietary-induced obesity. *Journal of Bone and Mineral Research*.

[73] Luu YK, Pessin JE, Judex S, Rubin J, Rubin CT (2009). Mechanical signals as a non-invasive means to influence mesenchymal stem cell fate, promoting bone and suppressing the fat phenotype. *BoneKEy Osteovision*.

[74] Kawaguchi H, Akune T, Yamaguchi M (2005). Distinct effects of PPAR*γ* insufficiency on bone marrow cells, osteoblasts, and osteoclastic cells. *Journal of Bone and Mineral Metabolism*.

[75] Liu J, Hoppman N, O'Connell JR (2012). A functional haplotype in EIF2AK3, an ER stress sensor, is associated with lower bone mineral density. *Journal of Bone and Mineral Research*.

[76] Taes YEC, Lapauw B, Vanbillemont G (2009). Fat mass is negatively associated with cortical bone size in young healthy male siblings. *Journal of Clinical Endocrinology and Metabolism*.

[77] Gong Y, Slee RB, Fukai N (2001). LDL receptor-related protein 5 (LRP5) affects bone accrual and eye development. *Cell*.

[78] Robling AG, Turner CH (2002). Mechanotransduction in bone: genetic effects on mechanosensitivity in mice. *Bone*.

[79] Robling AG, Turner CH (2009). Mechanical signaling for bone modeling and remodeling. *Critical Reviews in Eukaryotic Gene Expression*.

[80] Sawakami K, Robling AG, Ai M (2006). The Wnt co-receptor LRP5 is essential for skeletal mechanotransduction but not for the anabolic bone response to parathyroid hormone treatment. *The Journal of Biological Chemistry*.

[81] Kiel DP, Hannan MT, Barton BA (2010). Insights from the conduct of a device trial in older persons: low magnitude mechanical stimulation for musculoskeletal health. *Clinical Trials*.

[82] Little RD, Carulli JP, Del Mastro RG (2002). A mutation in the LDL receptor-related protein 5 gene results in the autosomal dominant high-bone-mass trait. *American Journal of Human Genetics*.

[83] Niziolek PJ, Warman ML, Robling AG (2012). Mechanotransduction in bone tissue: the A214V and G171V mutations in Lrp5 enhance load-induced osteogenesis in a surface-selective manner. *Bone*.

[84] Zhong Z, Zeng X, Ni J, Huang X (2013). Comparison of the biological response of osteoblasts after tension and compression. *European Journal of Orthodontics*.

[85] Lisse TS, Chun RF, Rieger S, Adams JS, Hewison M (2013). Vitamin D activation of functionally distinct regulatory miRNAs in primary human osteoblasts. *Journal of Bone and Mineral Research*.

[86] Sengul A, Santisuk R, Xing W, Kesavan C (2013). Systemic administration of an antagomir designed to inhibit mir-92, a regulator of angiogenesis, failed to modulate skeletal anabolic response to mechanical loading. *Physiological Research*.

[87] Lian JB, Stein GS, van Wijnen AJ (2012). MicroRNA control of bone formation and homeostasis. *Nature Reviews Endocrinology*.

[88] Luzi E, Marini F, Sala SC, Tognarini I, Galli G, Brandi ML (2008). Osteogenic differentiation of human adipose tissue-derived stem cells is modulated by the miR-26a targeting of the SMAD1 transcription factor. *Journal of Bone and Mineral Research*.

[89] Kapinas K, Kessler CB, Delany AM (2009). miR-29 suppression of osteonectin in osteoblasts: Regulation during differentiation and by canonical Wnt signaling. *Journal of Cellular Biochemistry*.

[90] Mizuno Y, Yagi K, Tokuzawa Y (2008). miR-125b inhibits osteoblastic differentiation by down-regulation of cell proliferation. *Biochemical and Biophysical Research Communications*.

[91] Zhang J, Fu W, He M (2011). MiRNA-20a promotes osteogenic differentiation of human mesenchymal stem cells by co-regulating BMP signaling. *RNA Biology*.

[92] Klein-Nulend J, Bacabac RG, Mullender MG (2005). Mechanobiology of bone tissue. *Pathologie Biologie*.

[93] Sikavitsas VI, Temenoff JS, Mikos AG (2001). Biomaterials and bone mechanotransduction. *Biomaterials*.

[94] Uddin SMZ, Cheng J, Lin W, Qin Y (2011). Low-intensity amplitude modulated ultrasound increases osteoblastic mineralization. *Cellular and Molecular Bioengineering*.

[95] Datta N, Pham QP, Sharma U, Sikavitsas VI, Jansen JA, Mikos AG (2006). In vitro generated extracellular matrix and fluid shear stress synergistically enhance 3D osteoblastic differentiation. *Proceedings of the National Academy of Sciences of the United States of America*.

[96] Mauney JR, Sjostorm S, Blumberg J (2004). Mechanical stimulation promotes osteogenic differentiation of human bone marrow stromal cells on 3-D partially demineralized bone scaffolds in vitro. *Calcified Tissue International*.

[97] Milan J, Planell JA, Lacroix D (2009). Computational modelling of the mechanical environment of osteogenesis within a polylactic acid-calcium phosphate glass scaffold. *Biomaterials*.

[98] Pioletti DP (2011). Biomechanics and tissue engineering. *Osteoporosis International*.

